# Smartphone-Based Device for Non-Invasive Heart-Rate Measurement of Chicken Embryos

**DOI:** 10.3390/s19224843

**Published:** 2019-11-06

**Authors:** Akkachai Phuphanin, Lawan Sampanporn, Boonsong Sutapun

**Affiliations:** 1 Institute of Research and Development, Suranaree University of Technology, 111 University Ave., Muang, Nakhon Ratchasima 30000, Thailand; akkachai@sut.ac.th (A.P.); lawan.s@sut.ac.th (L.S.); 2 School of Electronic Engineering, Institute of Engineering, Suranaree University of Technology, 111 University Ave., Muang, Nakhon Ratchasima 30000, Thailand

**Keywords:** heart rate measurement, chicken embryos, photoplethysmography, smartphone sensing

## Abstract

Heart rate (HR) is an important parameter in the study of the developmental physiology of chicken embryos and a crucial indicator of dead or live embryo grading in artificial incubation processes. A non-invasive HR measurement technique is required for long-term and routine HR assessment with minimal influence on embryo development. Accordingly, in this study, a non-invasive HR measurement technique of chicken embryos using a smartphone is demonstrated. The detection method of the proposed device is based on the photoplethysmography principle in which a smartphone camera is used for video recording, and the chicken embryonic HR is obtained from the recorded video images using a custom Android application. We used a smartphone to measure the embryonic HR of 60 native chicken eggs and found that it can measure the chicken embryonic HR from day 4 to day 20. The proposed smartphone HR device will be beneficial for scientific research and industrial applications. With internet connectivity, users can utilize their smartphone to measure the HR, display, share, and store the results.

## 1. Introduction

Heart rate (HR) is recognized as an important physiological parameter for studying the developmental physiology of chicken embryos, a widely used experimental animal model [1,2]. Several studies [3,4,5,6] have been conducted on the HR, HR patterns, and HR fluctuations of the developing chicken embryos which are affected by the environmental conditions in the artificial incubator such as temperature, humidity, and gas exchange, during the incubation period. Several measurement techniques for HR detection in chicken embryos have been developed in recent years, which can be categorized into three groups: invasive, semi-invasive, and non-invasive techniques. Invasive techniques, which involve catheterization for blood-pressure measurement [7], are acute processes and are not suitable for long-term measurement of embryonic HR.

Among the semi-invasive techniques, an electrocardiogram (ECG) has been widely used for chicken embryonic HR measurements [4,8,9,10,11] because of the ready availability of ECG equipment. The ECG technique utilizes metal electrodes inserted to a depth of 3–4 mm in the embryonic fluids through small holes made on the eggshell to measure the electrical signals generated by the heart. For long-term measurement, the metal electrodes are carefully positioned between the eggshell and the outer membrane, thereby minimizing damage to the egg membrane [12]. Furthermore, ECG can be used for HR detection of chick embryos during the second half of incubation [11,12]. In addition to ECG, pulse oximetry can be used to determine the HR of chick embryos without damaging the egg membrane, in the second half of the incubation period [13]; however, an area of diameter 1 mm must be removed on the eggshell, allowing red and near-infrared (NIR) lights to penetrate through the egg. Another semi-invasive technique has been developed based on video imaging and signal processing for continuous HR measurement of chicken embryos inside incubators [14]. However, this technique requires a large window opening on the eggshell, along with the application of plant oil to the membrane, allowing a universal serial bus (USB) camera fixed on top of the window to clearly see the blood vessels.

Early semi-invasive techniques involved the creation of holes on the eggshell or in the membrane. Removing the eggshell and the membrane affects the development of the embryos and increases the risk of bacterial infections in long-term measurements. In addition, eggshell removal is a delicate procedure that cannot be easily performed routinely and involves the risk of destruction of egg samples. Therefore, the semi-invasive techniques are impractical for daily or routine use.

Non-invasive HR detection techniques are based on the principles of acoustocardiography (ACG), ballistocardiography (BCG), and light absorption. The ACG technique [15] employs a microphone or a differential pressure sensor to measure the acoustic signals from the chicken embryo. To minimize the ambient acoustic noises, the egg sample must be placed in a tightly sealed vibration-free container. The BCG method [16,17] uses a laser displacement sensor or a phonograph cartridge to measure the minute movements of the eggshell caused by the recoil and impact of heart contraction. The HR signals obtained using this technique are normally contaminated by noises; therefore, the egg sample must be kept in an anti-vibration container or on an isolation table. The ACG and BCG techniques can be used for chicken embryonic HR measurement in the second half of incubation. Moreover, these methods require expensive equipment and an effective noise cancellation technique.

Embryonic HR measurement of chicken eggs based on light absorption variation due to pulsatile volumetric changes of blood vessels during systole and diastole of the cardiac cycle has gained great attention from researchers as a simple, accurate, and low-cost non-invasive method. Two different optical configurations, namely, reflection and transmission, are available in this method. In the optical reflection configuration, a near-infrared (NIR) light-emitting diode (LED) that serves as a light source is placed underneath the egg sample; the light undergoes multiple reflections inside the egg tissue and blood vessels, which are measured using a photodiode arranged perpendicular to the optical axes of the light source [18]. A commercial HR detector known as “buddy-digital egg monitor” [19] employs this optical configuration and has been used for HR measurements in chicken, turkey, and reptilian embryos [18,20,21]. In the transmission mode, a light source usually comprising NIR LEDs is placed underneath the sample, and the transmitted light is measured using a photodetector [22]. This approach is used for studying the HR patterns, HR variability, HR strength, and cardiac arrhythmia in chicken embryos during incubation [5,22,23]. Two approaches are available to extract the HR from time-varying waveforms: the time-domain peak detection method and the frequency-domain method. In the frequency-domain method, a pulse time waveform is converted into frequency domain using a fast-Fourier transform (FFT); furthermore, the HR is the dominant frequency because the main periodic component of the time-varying waveform originates from cardiac events. In addition, HR extraction using the frequency-domain method appears less vulnerable to noises induced by movements of the embryo, than that using the time-domain method [14]. The optical techniques can be used to measure the HR of chicken embryos on as early as day 6 of the incubation period.

The rapid growth of wireless telecommunication networks and billions of smartphone users present a remarkable opportunity for smartphone-based detection in biomedical applications, analytical devices, and portable scientific instruments. Most smartphones are equipped with an HR detection module that employs the contact-mode photoplethysmography (PPG) principle. The HR detection module comprises a dedicated LED light source with an integrated photodiode to detect the backscattered or reflected light from tissues. Several mobile applications for HR measurements have been developed and are commercially available. Instead of an extra HR detection module for HR measurement, a camera phone configured as a video imaging unit and an adjacent flashlight acting as a light source can be used to measure the HR [24]. In recent years, imaging PPG (IPPG), an emerging technique that uses a smartphone camera and image-processing techniques for non-contact and multipoint HR measurement, has been developed [25,26,27]. The IPPG reduces physical contact with the skin of the subject and has no restriction of subject movement; therefore, it enables remote monitoring and large-area blood perfusion mapping. Furthermore, the IPPG is a capable tool for personal health monitoring and home care. With advancements in smartphone hardware and software, expanding the capabilities of the smartphone-based PPG system for HR measurement of samples other than human subjects appears to be a rational solution.

In this study, we demonstrate the feasibility of a non-invasive and non-contact method to measure the HR of chicken embryos using a smartphone as the video imaging unit. We devised a sample holder with a light source that comprises several small LEDs to achieve even light-intensity illumination around the equator of the egg; in addition, it is suitable for dark-field illumination. The mechanical structure of the sample holder enables rejection of direct light from the light source and minimizes the ambient light interference. An Android application was developed for image acquisition and processing, as well as for embryonic HR extraction from the frequency spectrum. We used the smartphone device to measure the embryonic HR of 60 chicken eggs—54 fertile and 6 infertile eggs—and found that it can start measuring the chicken embryonic HR from day 4. The proposed smartphone HR detector may be advantageous for scientific research and industrial applications, including cardiovascular development research and precision poultry systems. Researchers can use their own smartphones to measure the HR of chicken embryos or other avian embryos. Moreover, with internet connectivity, users can use their smartphone to measure the HR, display, share, and store the results.

## 2. Materials and Methods

### 2.1. Materials

Experiments were conducted for a total of 60 white-eggshell chicken eggs from a flock of Thai indigenous (50% crossbred) Korat chickens [28] of which 54 were fertile and 6 were infertile. These eggs were brought from the poultry farm of Suranaree University of Technology. The eggs were collected daily between 2–3 pm, indicating that the collected eggs were laid only after 3 pm on the previous day of egg collection. All the eggs used in this study were collected on the same day. The eggs were then transferred from the poultry farm to the testing laboratory at around 5 pm; they were labelled and immediately placed in the incubator. In the experiments, the eggs were stored in a 60-egg custom-made laboratory incubator at 38.5 ℃ and 60% humidity and were automatically turned through an angle of 90° every hour. Day 0 refers to the day the eggs were collected from the poultry farm. From day 1 to day 20, the eggs were removed from the incubator once per day for daily HR measurement.

### 2.2. Setup

**Figure 1:**
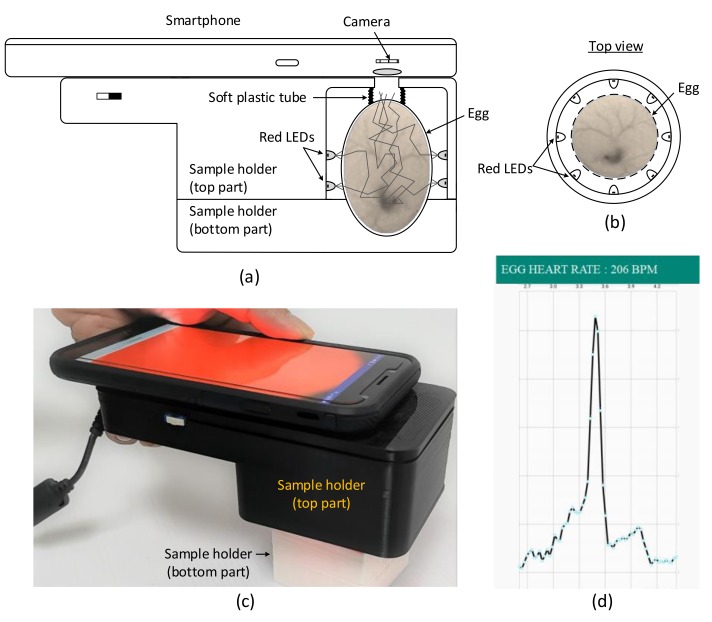
(**a**) Schematic of the setup for heart-rate (HR) measurement of chick embryos using a smartphone. (**b**) Arrangement of 16 red light-emitting diodes (LEDs) around the sample. (**c**) Prototype of the smartphone. (**d**) Screenshot of the smartphone Android application showing the frequency spectrum and the HR peak.

The setup for the HR measurement of chick embryos using smartphone comprises a smartphone, sample holder, and an LED light source, as shown in Figure 1. To measure the embryonic HR, an egg was removed from the incubator and placed in a 3D-printed sample holder, and a smartphone was placed on top of the holder and positioned such that the phone camera was aligned with the opening of the sample holder (Figure 1a). The egg was first placed in the bottom part of the holder and the top part was then gradually lowered and locked with the bottom part. The egg sample was held firmly but gently in the bottom part of the holder by a flexible black and soft plastic tube of diameter 2 cm, which was permanently fixed to the top part of the holder. In addition, the plastic tube served as a light shield to prevent direct illumination of the light source, but allowed the reflected or backscattered light from the embryonic tissue to reach the smartphone camera.

The top part of the sample holder consisted of an LED light source comprising 16 red LEDs (Avago, HLMP-EG, 670 nm, bandwidth 30 nm, maximum current 100 mA) arranged in 2 rows around the egg equator (Figure 1b). The LEDs were powered by a 24 V power supply and the current was set to 100 mA using a limiting resistor. Using several low-power LEDs provides an even illumination around the egg sample compared with using a single high-power and bright LED. Note that green LEDs, a widely used light source for IPPG-based HR detection in human subjects, is not suitable for use as a light source for HR measurement in chicken eggs because eggs naturally transmit a low level of light at this wavelength [29].

A smartphone camera (Galaxy J7, 1920 × 1080 pixels, Samsung, Seoul, South Korea) with a custom Android application placed on top of the sample holder (Figure 1c) was used to detect the light-intensity variation due to reflection or backscattering caused by the egg tissue. The blood flow increases in the blood vessels during systole, which increases the relative optical pathlength, resulting in low light intensity at the camera. The blood returns to the heart during diastole, causing decreased blood flow through the blood vessels, which leads to reduced relative optical pathlength, resulting in high light intensity at the camera. This pulsatile light-intensity fluctuation, known as the PPG waveform, synchronizes with the changes in blood volume during each heartbeat. A custom Android application transformed the PPG waveform to a frequency spectrum (Figure 1d) and calculated the heart rate.

It must be noted that the smartphone camera was placed at approximately 2 cm from the topmost surface of the egg, and an imaging area on the egg of about 3 cm^2^ was visible to the camera. The camera was unable to focus because of the close distance, resulting in blurred images. However, because the camera is intended to measure the total light intensity rather than obtaining the spatial image intensity or a high-contrast image of the egg, this problem is neglected. The manual focus setting of the camera was used in all experiments, because the camera attempts to find a focal point of the image for a few seconds in the autofocus mode, which results in varying imaging areas for different focal points.

### 2.3. Image Acquisition

The camera exposure time and the frame rate were fixed at 100 ms and 30 fps, respectively, in the experiments. The light transmitted through a fertile egg, particularly during the last stage of incubation, is relatively low, because of the growth of the chicken embryo body. The typical transmittance for an incubated fertile egg on day 0 is about 0.032%, which continuously decreases and reaches about 0.026% on day 16, for the wavelength of 670 nm [29]. Furthermore, the transmittance varies considerably between each egg because the eggs are of different sizes, colors, and eggshell thicknesses. To accommodate light intensity variations during the incubation, the camera was configured with the difference ISO setting, i.e., electronic gain, prior to each experiment, to enable the camera capture high intensity values. The ISOs for the first half and the second half of incubations were 100–200 and 200–400, respectively. Note that a large ISO produces higher camera noise than a lower one.

For the embryonic HR measurement, the eggs were removed from the incubator each day and placed in the sample holder, with the wide end of the egg facing upward, for video recording using the smartphone. The videos were recorded twice for 60 s at the frame rate of 30 fps, thus obtaining a total of 1800 frames. The video files recorded first were analyzed using the custom Android application. Similarly, the second video files were recorded for 60 s for each egg in the MPEG-4 file format and were transferred to a computer for further analysis.

### 2.4. Android Application Development

To extract the HR from the recorded videos, a custom program was developed using the Android developer tools (Android Studio 3.2.1, Google, Mountain View, CA, USA). The first step was to convert the video images from the YUV420 color space to the RGB color space using the available formulas [30]. Only the pixel values in the R channel were used for HR extraction. Next, the PPG waveform, which is the spatial average intensity of all red pixels in the entire imaging area plotted as a function of time, was obtained and stored in the memory. Because the images were recorded at 30 fps for 60 s, a total of 1800 data points were recorded, and each data point had a time difference of 1/30 s. The raw PPG waveforms obtained from the fertile eggs had relatively small-amplitude signals riding on large direct current (DC) and low-frequency components due to the moving artifacts; therefore, the HR periodical signals may be not clearly visible.

To suppress the moving artifacts, the raw PPG waveforms were applied by a detrending operation using the weighed moving average technique (23-term Henderson method [31]). The processed PPG signals were then analyzed using the FFT technique (Apache Commons Math 3.6.1 API, The Apache Software Foundation, Forest Hill, MD, USA) using the number of samples (N = 1024), following which a flat-top window was applied. The chicken embryonic HR is normally in the range 2–5 Hz, which is well below the Nyquist frequency of this system, which is 30/2 = 15 Hz. Next, a bandpass filter of 0.5–10 Hz was applied to remove the DC and low-frequency components and the high-frequency noises that may be present in the power spectrum. The final step was the extraction of HR rate, which is the frequency of the highest peak in the filtered spectrum, using a peak detection technique.

In early days of incubation, the cardiac activity signal strength is lower than the DC signal component; thus, the amplitude of the HR peak in the frequency domain may be obscured by noise. To differentiate the HR peak from noise, we considered the dominant peak as the HR peak only if the height of the dominant peak is three times larger than that of the noise floor level. If no such dominant peak was found in the first set of the PPG waveform, the next 1024 points of data with 50% overlapping between the consecutive windows were used to calculate the power spectrum; further, the dominant peak was recalculated and compared with the noise floor level until the previous condition was satisfied.

### 2.5. Image Processing and Heart-Rate (HR) Calculation Using a Computer-Based Software

**Figure 2:**
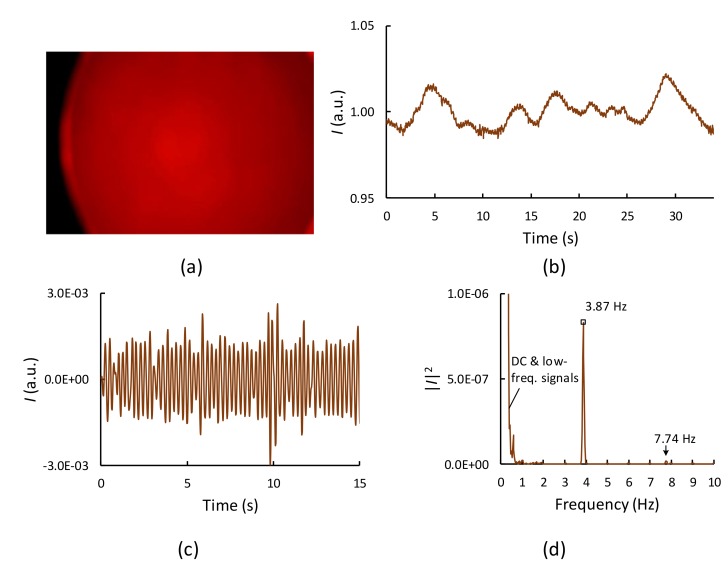
Embryonic heart rate extraction from a recorded video file. (**a**) Typical image acquired from a fertile egg using the smartphone setup, on day 13 of incubation. (**b**) Normalized photoplethysmography (PPG) signal extracted from the recorded video file. (**c**) Processed PPG waveform after the detrending and filtering methods were applied. (**d**) Corresponding power spectrum in the range 0–10 Hz (without applying bandpass filtering).

**Figure 3:**
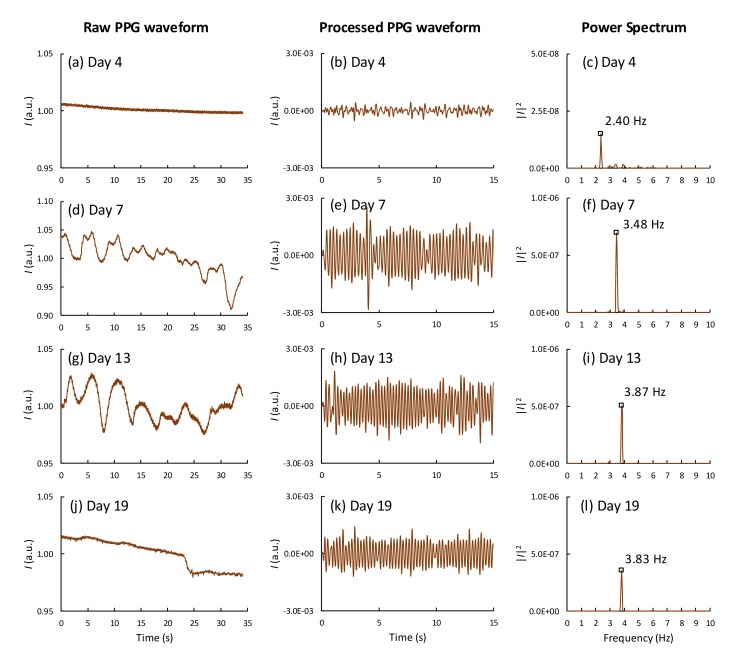
PPG waveforms and their corresponding power spectra of the processed waveforms obtained from a fertile and live egg during incubation on (**a**–**c**) day 4, (**d**–**f**) day 7, (**g**–**i**) day 13, and (**j**–**l**) day 19.

**Figure 4:**
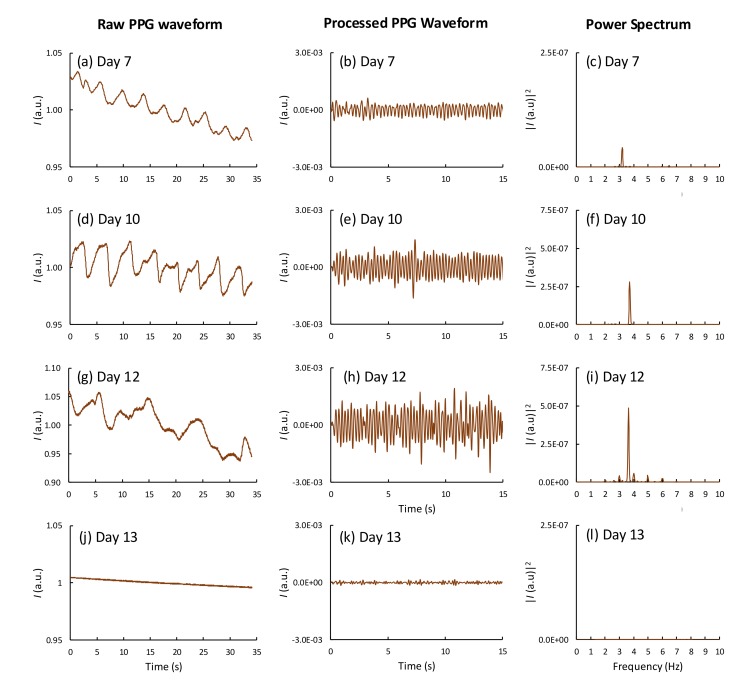
PPG waveforms and the corresponding power spectra obtained from an egg whose embryo died on day 13 during incubation on (**a**–**c**) day 7, (**d**–**f**) day 10, (**g**–**i**) day 12, and (**j**–**l**) day 13.

For data presentation (Figure 2, Figure 3 and Figure 4, Figures S1 and S2) and verification of the Android application of the HR calculation algorithm, the recorded video files were transferred to a computer for image processing and HR calculation using a custom program developed based on LabVIEW 2017 (National Instruments, Austin, TX, USA). First, the stored MPEG-4 file was converted into an AVI file using a video file converter (AVS video converter 8.3) before importing it to the LabVIEW program. Next, the color format of the recorded video images was converted to RGB, and only the R-pixel data were used. The PPG waveform was calculated from the whole image. To minimize the background noises and suppress the moving artifacts, the raw PPG waveform was detrended using the 23-term Henderson moving average method, and a bandpass filter was subsequently applied at 0.5–10.0 Hz. The processed PPG waveform was then analyzed using the FFT technique using the number of samples of 1024 data points, following which a flat-top window was applied. The moving average, bandpass filter, and the FFT functions are available in the LabVIEW libraries. The peak detection technique was employed to identify the location of the dominant peak in the power spectrum, and the embryonic HR was calculated using the same condition described in the previous section. We found that the HR results obtained from the online Android application and the offline LabVIEW software are linearly correlated (*r* = 0.99) from day 4 to day 20.

### 2.6. Experimental

Each of the 60 eggs (54 fertile and 6 infertile) was removed from the incubator for HR measurement every day at 1–2 pm, from day 1 to day 20. The fertile and live eggs were verified by successful hatching, and the dead and infertile eggs were broken and verified by visual observation at the end of incubation. Out of the 54 fertile eggs, 42 hatched successfully, 6 were alive but did not hatch on day 21, and 6 eggs died during the incubation period. These eggs died between days 9–13, as observed from the daily HR signals.

For the embryonic HR measurement, the eggs were removed from the incubator (in batches of 9–10 eggs) every day and each egg was placed in the setup for video recording by the smartphone. The videos were recorded twice at 30 fps for 60 s for each egg, thus obtaining a total of 1800 frames. The measurement for each batch of samples lasted for about 30 min, and the eggs were placed back in the incubator after the measurement. All the experiments were conducted at room temperature (25–27 ℃).

## 3. Results

Figure 2a shows the image of a fertile egg on day 13 obtained using the smartphone. The image of the egg is blurred because the smartphone camera was placed very close to the sample and was thus out of focus. The PPG waveform, which is the light intensity (*I*) spatially averaged over all red pixels in the whole picture area plotted as a function of time, obtained from this egg is shown in Figure 2b. Note that the measured intensity was normalized with the mean intensity value calculated for the 60-s period. The raw PPG waveform contains a small-amplitude cardiac signal, a low-frequency signal caused by the embryo movement, and a DC component. After applying the detrending and bandpass filtering, the PPG signal apparently showed a periodic cardiac signal (Figure 2c). The corresponding power spectra (Figure 2d) when using the raw PPG waveform (Figure 2a) as an input clearly indicate a dominant peak at 3.87 Hz (232 beats per minute (BPM)), which represents the embryonic HR signal. In addition, the second harmonic at 7.74 Hz and the large low-frequency component caused by embryo movements, typically less than 1 Hz, were found in the frequency spectrum, but were filtered out later when a bandpass filter of 0.5–10 Hz was applied.

Figure 3 shows the development of PPG waveforms and their corresponding power spectra obtained from one of the fertile eggs at different stages of incubation using the smartphone setup. For this egg (sample no. 14), the cardiac activity was observed from day 4 of incubation (see Table S1). On day 4, the time-varying cardiac signal had a very small amplitude and was barely visible; however, the corresponding power spectrum clearly showed the HR at 2.40 Hz (144 BPM). The HR and the HR strength, which is the amplitude of the PPG signal or the area under the curve of the HR peak in the power spectral, increased over time till days 10–12 and reached a plateau during days 13–18. On days 19–20, the PPG signal strength was significantly reduced but was still observable. The embryo movements appeared normally in the PPG signals as slow time-varying signals, or in the power spectrum as low-frequency components below 1 Hz. To extract the HR from the time-varying waveforms, the frequency-domain method using FFT is more suitable than the time-domain method at the early stages of incubation, when the HR signal strength is relatively low. For comparison, the PPG waveforms and the power spectra of an infertile egg are shown in Figure S1. Only a DC component was observed in the corresponding power spectrum obtained from the infertile eggs.

Out of the 54 fertile eggs, 6 embryos died during incubation. An analysis of the daily PPG signals and power spectra revealed that 3 eggs died on day 9, 2 on day 10, and another on day 13. Figure 4 shows the PPG waveforms and the corresponding power spectra obtained for an egg whose embryo died on day 13 (sample no. 47, Table S1). The cardiac activity of this egg was observed for days 6–12. On day 13, no heart beat was detected in both the time-varying signals and the power spectrum. To verify this result, we monitored the HR of the eggs every day until day 21 and found no cardiac signal from the dead eggs. Subsequently, the dead eggs were broken on day 21 for visual inspection (see Figure S2).

Table 1 lists the mean HR obtained on each day of incubation, and the standard deviation for 48 fertile and live eggs obtained using the proposed smartphone setup. The HR can be measured from as early as day 4 for some eggs. The average HR increases from 150 BPM on day 4 to 222 BPM on day 10. For days 11–19, the HR reaches a plateau and fluctuates between 206–231 BPM. On day 20, the HR drops to 180 BPM with a large standard deviation of 49 BPM. Furthermore, the HR results obtained daily for each egg are listed in Table S1.

**Figure 5:**
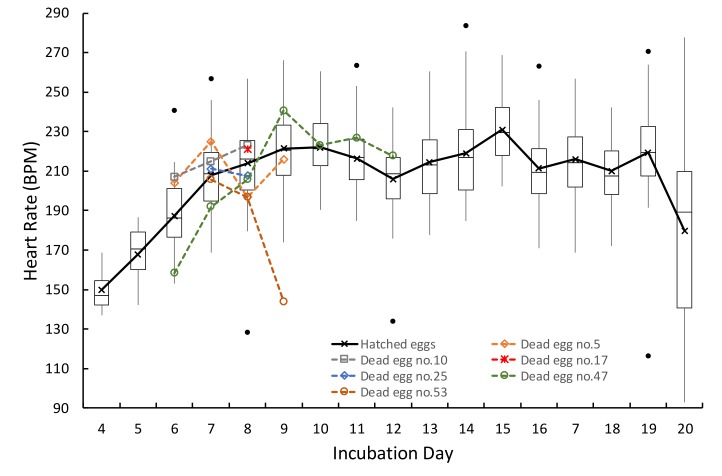
Mean embryonic HR obtained during incubation, from fertile and live eggs using the proposed smartphone device setup and the custom Android application. The daily HRs obtained from 6 dead embryos are given for comparison.

Figure 5 plots the mean HR of the fertile and live eggs as a function of the day of incubation; the daily HRs of 6 dead eggs are shown in the same plot. The results indicate no apparent difference in the HR patterns between live and dead eggs. However, to find a correlation between the health status of chicken embryos and the embryonic HR pattern using appropriate statistical analysis, more data from dead eggs are required.

## 4. Discussion

The main objective of this work is to demonstrate the feasibility of using a smartphone as portable scientific equipment for non-invasive chicken embryonic HR measurement. The detection method of the proposed device is based on the PPG principle in which a smartphone camera is used for video recording and the embryonic HR is extracted from the recorded videos using a custom Android application. A total of 60 chicken eggs were tested for the HR measurement during days 1–20.

The measured mean HRs of chicken embryos during the incubation period using the smartphone device show a similar pattern to the results reported by other works [8,18,21]. Our device can be used to measure the HR of chicken embryos of all fertile eggs on day 7 and as early as day 4 for some eggs. During the second half of incubation, the HR reaches a plateau of 206–231 BPM. Lierz et al. [18] reported a similar HR pattern using a digital egg monitor to measure the HR of chicken embryos. They reported higher HR values of 241–273 BPM during the second half of the incubation period compared with our results. Similarly, Noiva et al. [21] used a digital egg monitor to measure the HR of chicken embryos and reported HR values of 218–234 BPM in the second half of incubation, which is comparable to our results. The discrepancy in HR values between these works possibly depends on the amount of time the eggs were kept out of the incubator during the experiment. In our work, as well as in [21], the eggs were taken out of incubators in batches for HR measurements, and were left at room temperature for several minutes before placing them back in the incubators. However, in [18], the eggs were taken out of the incubator in individual succession for measurement, and were immediately returned to the incubator after the experiment. Previous studies indicate that the HR of the chicken embryos drops by about 30–50 BPM when left at room temperature for 20–30 min [18,32]. The mean embryonic HR measurements using a NIR photodetection unit yielded a BPM of 228–290 during the second half of incubation when the sample eggs were left out of the incubators for a short time [23] or when left in a warm electric heating blanket [22] during the experiment. These results suggest that the proposed smartphone device method is suitable for HR measurement of chicken embryos; in addition, they offer similar HR results as previous reports on non-invasive optical-based HR measurement of chicken embryos, such as the digital egg monitor [19] and the NIR photodetection technique [22], provided that the testing conditions and the environment are the same. Moreover, the proposed smartphone device offers additional advantages, including a low-cost hardware platform, network connectivity, simple data storing and sharing, and software updates.

Previous works employed NIR LEDs of wavelength 800–850 nm as the preferred light source in designing an HR measurement device for chicken eggs based on the PPG principle. The NIR LEDs offer several advantages such as highest transmittance through the egg, low power consumption, low heat generation, and low cost. However, they are not suitable for smartphone devices because a smartphone camera has integrated optical filters that allow only lights in the red, green, and blue regions to pass through; in addition, it has a NIR cut off filter to block any unwanted signal that distorts the color images. Our past experiences indicate that the signal to noise ratio is relatively low when NIR LEDs are used with a smartphone camera. In our work, therefore, we chose red LEDs as the light source because they offer a higher signal-to-noise ratio than the NIR LEDs. A disadvantage of using a red LED as the light source is that the egg transmittance is relatively low. The typical transmittance for incubated fertile eggs on day 16 is approximately 0.02%–0.03% at the wavelength of 670 nm [29], and is much lower on days 19–20. We found that even when using an optical set up in the transmission arrangement, i.e., red LED light source underneath the egg sample and the camera above the egg, the signal-to-noise ratio is low. To increase the signal to noise ratio, we chose a different optical arrangement of placing several small red LEDs around the equator of the sample egg and the camera above the egg. With the proposed optical arrangement, a higher fraction of the incident light reaches the camera than with a transmission arrangement; therefore, the HR signals are stronger at later times during the incubation period. The signal-to-noise ratio obtained for this optical arrangement employing 16 red LEDs is relatively high, and we could measure the HR for most eggs during days 19–20. In our experience, by using 6–8 red LEDs arranged in the same configuration, the HR was detected during the first half of the incubation period but not during the second half. Increasing the number of red LEDs improved the signal-to-noise ratio, but the power consumption was higher. We believe that using 16 red LEDs is a reasonable trade-off between signal-to-noise ratio and power consumption. This optical arrangement must be useful for HR measurement of large eggs such as duck and goose eggs, whose transmittance is very low.

Furthermore, in our study, the mortality rate of the embryos during incubation is relatively higher at 11% (6 dead eggs out of 54 fertile eggs) and the hatchability rate is lower at 87.5% (42 successfully hatched eggs and 6 unhatched eggs out of 48 live eggs) than those obtained for industrial settings. Five embryos died during days 9–10 and one on day 13. We attribute the high mortality and low hatchability rates in our experiments to the daily exposure of the eggs to room temperature for 20–30 min since day 1. Another reason is that the environment of the custom-made incubator is not as well-controlled as that in industrial incubators. Reducing the experiment time and employing a temperature-controlled sample holder may reduce the mortality rate and improve the hatchability rate. These results demonstrate the practicality of the proposed non-invasive HR monitoring device to study the mortality rate at each stage of incubation, which can be used to improve the incubation conditions.

The accuracy of the HR measurements of chicken embryos was reported by Lewin et al. [13] using an optical technique in comparison with the HR measured by the ECG method, which is widely accepted as the reference for HR measurement. They measured the HR of a chicken embryo on day 18 using both an optical technique and the ECG method simultaneously for 10 min and found that no significant difference exists between the two measurements. No other reports on the accuracy of measurement of the optical methods, for HR measurement of chicken embryos, are available in the literature. Thus, it can be assumed that the accuracy of HR measurement using optical techniques such as PPG and IPPG has been widely verified for human subjects, and the results can be readily applied for HR measurements of chicken embryos. Nevertheless, further experiments to determine the accuracy of the embryonic HR measurements using a smartphone device for different stages of incubation are required in the future.

## 5. Conclusions

This study demonstrated the feasibility of using a smartphone for non-invasive heart rate measurement of chicken embryos during incubation. To measure the embryonic heart rate, the egg sample was placed in a sample holder containing a red LED module arranged around the equator of the sample. The smartphone camera positioned on top of the sample holder was configured as the light detection unit which measures the reflected or backscattered light from the egg tissues and blood vessels. An Android application was developed for image acquisition, signal processing, and heart-rate extraction from the recorded time waveforms using a fast-Fourier transform. The proposed smartphone device setup measured the heart rate of the chicken embryos from day 4 to day 20; the mean heart rate variation pattern agreed well with the results of previous works. Using a smartphone for heart rate measurement allows the users to conveniently display and transfer the results and organize the data through the Internet. The non-invasive nature of this device is suitable for routine heart rate measurement of chicken embryos, as well as other avian embryos, without opening the eggshell, resulting in minimal influence on embryo growth. The proposed smartphone-based heart-rate device can be an advantageous tool in developmental physiology research, breeding development programs, and industrial poultry production.

## Figures and Tables

**Table 1:** Mean chicken embryonic HR, standard deviation, median and interquartile range of fertile and live eggs, throughout the incubation measured using the proposed smartphone device setup and the custom Android application. All parameters are given in BPM.

**Day**	4	5	6	7	8	9	10	11	12	13	14	15	16	17	18	19	20
**Number of Eggs**	4	13	34	48	48	48	48	48	48	48	48	48	47	48	48	48	38
**HR**	150	168	187	208	214	221	222	216	206	215	219	231	211	216	210	219	180
**SD**	14	14	19	18	21	19	17	17	19	20	22	16	18	21	18	24	49
**Median**	147	170	186	209	216	221	221	217	209	213	217	230	209	214	208	220	189
**25th Percentile**	142	160	176	195	200	208	213	206	196	198	200	218	199	202	198	208	141
**75th Percentile**	155	179	201	220	225	233	234	226	217	226	231	242	222	227	220	233	210

## Supplementary Materials

The following are available online at https://www.mdpi.com/1424-8220/19/22/4843/s1, Figure S1. PPG waveforms and power spectrum obtained for an infertile egg on day 9 (sample no. 21) (Table S1) using the smartphone HR device. (a) Raw PPG waveform. (b) PPG waveform after detrending and filtering. (c) Power spectra of (a) showing no cardiac signal in the range 2–5 Hz. (d) Picture of a broken egg on day 21 showing that the egg was infertile. Figure S2. Power spectra for all 6 dead eggs (No. 5, 10, 17, 25, 47, 53) (Table S1) obtained on the day before and the day each embryo died, during incubation. Pictures of the eggs broken on day 21 were used to confirm that the eggs were fertile, but the embryos died during incubation. Table S1. Measured heart rates using the proposed smartphone setup for 60 chicken eggs during days 1–20 of incubation.
