# Supplementary material for: Smartphone-Based Device for Non-Invasive Heart-Rate Measurement of Chicken Embryos
Source: Sensors (Basel). 2019 Nov 6;19(22):4843. doi: 10.3390/s19224843 (PMC6891743; doi:10.3390/s19224843)
Supplement: Supplementary file 1 [file sensors-19-04843-s001.pdf]

Supplementary Materials

Smartphone-Based Device for Noninvasive Heart Rate Measurement of Chicken Embryos

Akkachai Phuphanin [1], **Lawan Sampanporn** [1] **and Boonsong Sutapun** [2,*]

[1] Institute of Research and Development, Suranaree University of Technology, 111 University Ave., Muang, Nakhon Ratchasima 30000, Thailand

[2] School of Electronic Engineering, Institute of Engineering, Suranaree University of Technology, 111 University Ave., Muang, Nakhon Ratchasima 30000, Thailand

* Correspondence: boonsong@sut.ac.th

S1. Results obtained for an infertile egg

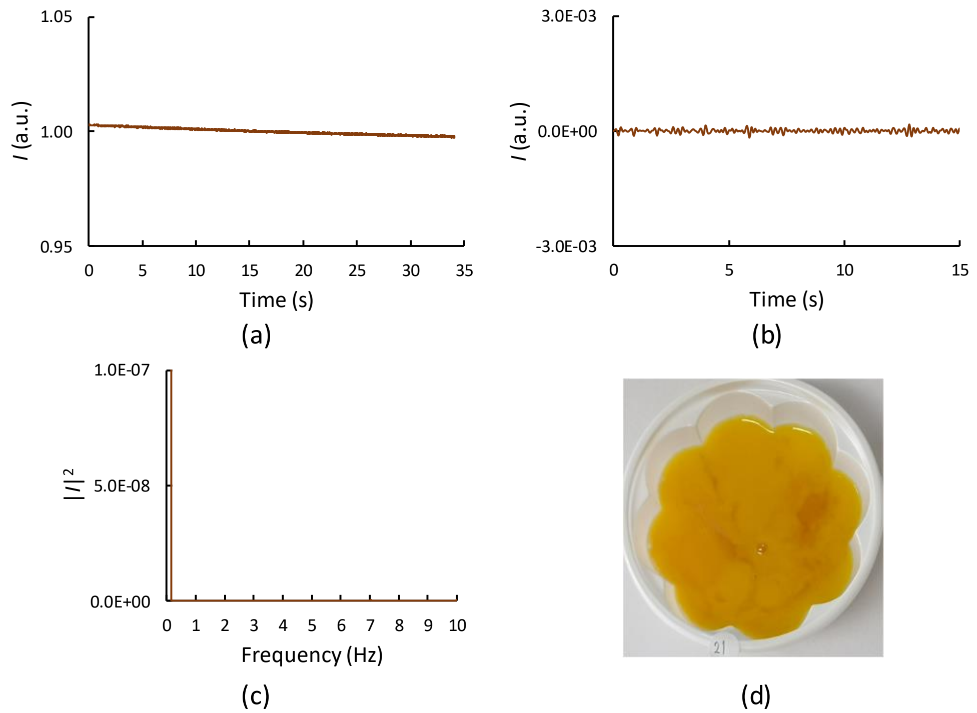

Figure S1. PPG waveforms and power spectrum obtained for an infertile egg on day 9 (sample no. 21) (Table S1) using the smartphone HR device. **(a)** Raw PPG waveform. **(b)** PPG waveform after detrending and filtering. **(c)** Power spectra of **(a)** showing no cardiac signal in the range 2–5 Hz. **(d)** Picture of a broken egg on day 21 showing that the egg was infertile.

S2. Results obtained for dead eggs

Out of the 54 fertile eggs, the embryos of 6 eggs died during incubation. Further, 3 embryos died on day 9, 2 on day 10, and 1 on day 13.

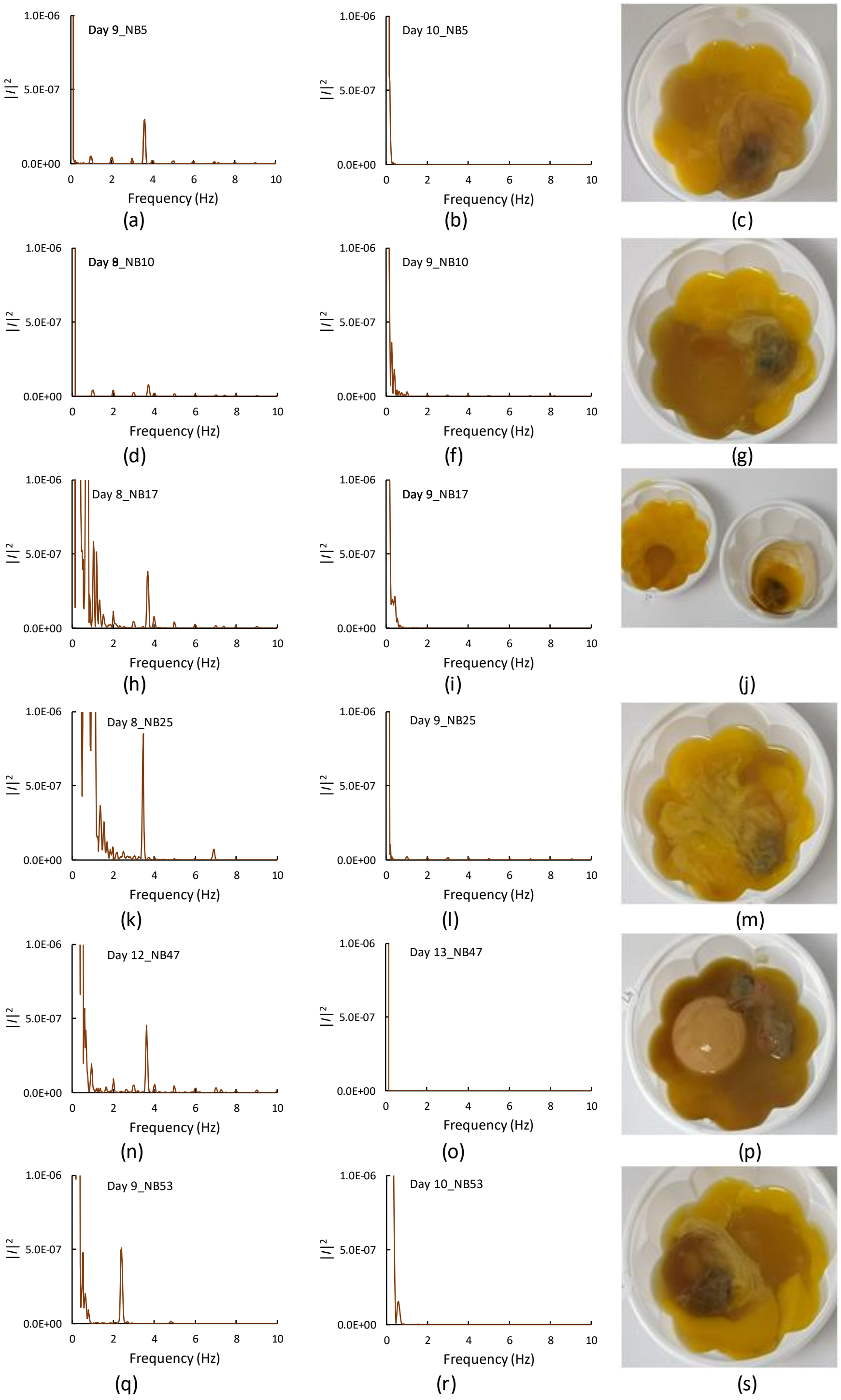

Figure S2. Power spectra for all 6 dead eggs (No. 5, 10, 17, 25, 47, 53) (Table S1) obtained on the day before and the day each embryo died, during incubation. Pictures of the eggs broken on day 21 were used to confirm that the eggs were fertile, but the embryos died during incubation.

S3. Heart rate of 60 individual eggs during incubation

Table S1. Measured heart rates using the proposed smartphone setup for 60 chicken eggs during days 1–20 of incubation.

Egg No.	Heart Rate (BPM)																				
	Day 1	Day 2	Day 3	Day 4	Day 5	Day 6	Day 7	Day 8	Day 9	Day 10	Day 11	Day 12	Day 13	Day 14	Day 15	Day 16	Day 17	Day 18	Day 19	Day 20	Notes
**1**	-	-	-	-	-	204	219	179	232	221	216	225	223	215	248	263	223	239	230	168	Hatched
**2**	-	-	-	-	-	192	218	238	233	236	238	230	222	211	239	220	211	206	264	95	Hatched
**3**	-	-	-	-	-	199	215	220	209	209	204	202	206	191	225	192	192	190	229	202	Hatched
**4**	-	-	-	-	178	196	209	213	220	206	211	218	211	206	229	208	202	220	227	151	Hatched
**5**	-	-	-	-	-	204	225	197	216	-	-	-	-	-	-	-	-	-	-	-	*Dead*
**6**	-	-	-	-	-	182	202	225	220	204	206	202	193	192	221	199	197	190	211	100	Hatched
**7**	-	-	-	-	-	178	197	211	204	197	204	182	188	190	210	202	190	193	191	Hatched	
**8**	-	-	-	-	-	179	195	223	212	208	208	197	197	200	218	206	197	193	223	122	Hatched
**9**	-	-	-	-	-	207	179	185	206	190	188	181	178	185	210	171	169	172	209	148	Hatched
**10**	-	-	-	-	-	207	215	223	-	-	-	-	-	-	-	-	-	-	-	-	*Dead*
**11**	-	-	-	-	-	204	220	128	215	238	236	238	257	253	253	230	257	238	241	Hatched	
**12**	-	-	-	-	-	-	185	213	211	227	227	227	230	230	244	215	244	220	211	258	Hatched
**13**	-	-	-	-	-	185	197	200	206	215	210	212	208	211	218	195	169	179	116	195	Hatched
**14**	-	-	-	137	167	194	217	242	227	221	220	221	232	233	235	213	227	234	209	Hatched	
**15**	-	-	-	150	160	206	220	215	215	234	225	216	223	230	242	232	236	208	250	Hatched	
**16**	-	-	-	-	-	-	-	-	-	-	-	-	-	-	-	-	-	-	-	-	*Infertile*
**17**	-	-	-	-	-	-	-	221	-	-	-	-	-	-	-	-	-	-	-	-	*Dead*
**18**	-	-	-	-	-	170	181	192	187	192	188	182	193	190	202	193	216	197	200	216	Hatched
**19**	-	-	-	169	181	241	230	238	244	236	236	230	260	241	269	236	246	241	239	Hatched	
**20**	-	-	-	-	179	215	257	257	266	260	253	242	251	244	265	238	246	242	230	Hatched	
**21**	-	-	-	-	-	-	-	-	-	-	-	-	-	-	-	-	-	-	-	-	Infertile
**22**	-	-	-	-	-		230	199	248	221	234	217	225	220	225	216	225	229	259	168	Hatched
**23**	-	-	-	-	176	195	236	213	230	230	220	215	220	213	225	214	221	218	239	Hatched	
**24**	-	-	-	-	-	-	-	-	-	-	-	-	-	-	-	-	-	-	-	-	*Infertile*
**25**	-	-	-	-	-	-	211	208	-	-	-	-	-	-	-	-	-	-	-	-	*Dead*
**26**	-	-	-	-	-	193	221	238	234	220	225	206	218	208	227	202	230	218	241	242	Hatched
**27**	-	-	-	-	-	-	218	227	239	223	216	197	202	200	211	190	199	195	209	137	Hatched
**28**	-	-	-	-	144	202	246	244	263	238	238	225	244	283	238	246	255	234	271	Hatched	
**29**	-	-	-	-	170	209	232	223	238	246	238	239	241	271	264	230	239	232	234	278	Hatched
**30**	-	-	-	-	-	181	220	225	227	221	208	205	209	236	232	209	229	218	229	100	Hatched
**31**	-	-	-	-	187	179	208	197	211	229	222	212	212	241	232	209	206	208	216	211	*Did not hatch*
**32**	-	-	-	-	-	190	218	229	229	230	218	211	223	250	251	220	225	214	214	206	Hatched
**33**	-	-	-	-	-	-	200	209	218	221	197	204	212	227	230	206	216	203	211	208	Hatched
**34**	-	-	-	-	-	172	209	220	220	202	199	199	200	221	223	197	202	185	212	229	Hatched
**35**	-	-	-	-	-	167	197	209	225	204	196	204	202	206	229	199	195	204	197	202	Hatched
**36**	-	-	-	-	-	153	188	223	221	191	193	192	193	195	227	200	202	220	203	153	Hatched
**37**	-	-	-	-	-	-	217	220	238	238	226	220	214	229	232	223	242	214	223	197	*Did not hatch*
**38**	-	-	-	-	-	-	204	227	174	225	212	212	188	191	262	223	212	209	236	187	Hatched
**39**	-	-	-	-	-	188	208	220	244	239	228	212	209	220	227	216	212	214	203	191	Hatched
**40**	-	-	-	-	-	-	223	236	242	232	229	214	221	241	214	209	204	223	202	*Did not hatch*	
**41**	-	-	-	-	-	179	202	229	206	234	220	221	220	220	246	211	223	208	230	241	Hatched
**42**	-	-	-	-	-	-	169	188	200	221	206	209	199	197	236	188	200	195	234	179	Hatched
**43**	-	-	-	-	-	160	190	200	190	216	206	193	195	206	209	199	203	204	208	195	Hatched
**44**	-	-	-	-	-	-	202	200	202	218	212	208	209	221	204	218	221	199	234	139	Hatched
**45**	-	-	-	-	-	159	186	191	200	193	185	185	187	195	232	181	184	172	212	211	Hatched
**46**	-	-	-	-	-	181	221	214	229	232	263	182	232	225	242	239	253	242	225	136	Hatched
**47**	-	-	-	-	-	158	192	206	241	223	227	218	-	-	-	-	-	-	-	-	*Dead*
**48**	-	-	-	-	-	-	-	-	-	-	-	-	-	-	-	-	-	-	-	-	*Infertile*
**49**	-	-	-	-	-	-	193	212	211	214	220	134	182	216	246	230	250	232	229	251	*Did not hatch*
**50**	-	-	-	-	142	163	195	223	233	230	218	209	218	212	225	223	225	212	199	100	Hatched
**51**	-	-	-	-	-	-	206	218	204	216	199	216	198	199	216	193	200	190	209	133	Hatched
**52**	-	-	-	-	-	-	184	200	191	195	197	176	197	197	209	192	190	199	193	183	Hatched
**53**	-	-	-	-	-	-	206	197	144	-	-	-	-	-	-	-	-	-	-	-	*Dead*
**54**	-	-	-	-	-	-	-	-	-	-	-	-	-	-	-	-	-	-	-	-	*Infertile*
**55**	-	-	-	-	179	206	222	221	233	255	206	200	242	250	244	211	227	200	232	227	Hatched
**56**	-	-	-	-	-	-	-	-	-	-	-	-	-	-	-	-	-	-	-	-	*Infertile*
**57**	-	-	-	-	-	-	220	236	252	230	209	188	234	230	230	220	211	206	208	93	Hatched
**58**	-	-	-	144	163	176	209	200	221	242	225	197	232	236	232	209	223	216	230	191	*Did not hatch*
**59**	-	-	-	-	156	165	190	202	218	218	223	188	216	218	218	*	211	202	206	*Did not hatch*	
**60**	-	-	-	-	-	-	179	195	229	239	233	190	229	216	214	199	211	202	199	188	Hatched

* Data unavailable due to large unknown noises
